# Evaluation of the phytochemical content, in vitro antioxidant capacity, biochemical and histological effects of *Dennettia tripetala* fruits in healthy rats

**DOI:** 10.1002/fsn3.792

**Published:** 2018-11-19

**Authors:** Sylvia O. Omage, Noghayin E. J. Orhue, Kingsley Omage

**Affiliations:** ^1^ Department of Biochemistry University of Benin Benin City Nigeria; ^2^ Department of Biochemistry School of Basic Medical Sciences College of Health Sciences Igbinedion University Okada Okada Edo state Nigeria

**Keywords:** antioxidant, aqueous extract, *Dennettia tripetala*, ethanolic extract, phytochemistry, toxicology

## Abstract

We estimated the content of specific phytochemicals and in vitro antioxidant properties of the powder, aqueous, and ethanolic extracts of ripe *Dennettia tripetala* fruits. We also tested the biochemical and histological effects of these fruit extracts on healthy rats. Aqueous and ethanolic extracts were prepared from the powder of ripe *D. tripetala* fruits, and standard phytochemical methods were used to evaluate its phytochemical content and antioxidant properties. Eighteen rats were randomized into three groups, one of which served as control, while the second and third groups received the aqueous and ethanolic extracts of *D. tripetala* fruits, respectively, at a dose of 1,000 mg/kg bw daily for 28 days. Our results show that the powder as well as the aqueous and ethanolic extracts of ripe *D. tripetala* fruits contains phenols, flavonoids, saponins, tannins, and alkaloids. The plant powder as well as both extracts scavenged DPPH and hydrogen peroxide as well as reduced ferric ions. The extracts of *D. tripetal*a fruits did not alter liver marker enzymes or serum protein profile of the rats. The extracts also did not alter the serum concentration of urea and creatinine and the antioxidant enzyme activity and lipid peroxidation levels in the liver but altered that of the kidney. The extracts altered the serum and liver lipid profile but not to any significant extents. Also, the extracts caused minimal congestion to the centrioles of the liver but were not in any other way toxic to the liver, kidney, or heart of the rats. Our results point to the fact that the fruits of *D. tripetala* possess phytochemicals with medicinal properties and are well tolerated by rats.

## 1 INTRODUCTION

A good percentage of drugs in use today are derived from plants or contain synthetic analogues built on prototype compounds isolated from plants (Soetan & Aiyelaagbe, 2009). It has been observed that many people especially in less developed countries rely on medicinal plants for the management and prevention of ailments (Soetan & Aiyelaagbe, 2009). Although these medicinal plants when consumed raw or in form of crude extracts elicit potent medicinal effects, they can also be toxic when consumed in high doses or for prolonged periods. There have been reports of kidney problems as well as other toxic effects in people consuming high doses of crude medicinal plant extracts (Bwititi, Musabayane, & Nhachi, 2000; Ijeh & Agbo, 2006). There is therefore a need for evaluation of the safety of medicinal plants.

*Dennettia tripetala* or Pepperfruit is a medicinal plant that grows in Nigeria and some parts of West Africa. It is a member of the family of *Annonaceae*. It is also known as “Ako,” “Mmimi,” or “Ata Igbere” by the Edo‐, Ibo‐, and Yoruba‐speaking people of Nigeria, respectively. It is consumed not just for its spicy taste but for its medicinal value (Iseghohi, 2015). It is used to manage cough, sore throat, fever, nausea, and other ailments. It contains antioxidant vitamins (Ihemeje, Ojinnaka, Obi, & Ekwe, 2013; Okolie, Falodun, & Davids, 2014) and several phytochemicals that have been found to elicit medicinal effects (Adedayo, Oboh, & Akindahunsi, 2010; Egharevba & Idah, 2015; Elekwa, Okereke, & Chukwudomo, 2011; López‐Martín, Anam, Boira, Sanz, & Blázquez, 2002). *D. tripetala* has been shown to elicit analgesic and anti‐inflammatory properties in rodents (Oyemitan, Iwalewa, Akanmu, & Olugbade, 2008). The plant also elicits antihyperglycemic effects in rats (Anaga & Asuzu, 2010) as well as hepatoprotective and nephroprotective effects in rats poisoned with CCl_4_ (Iseghohi & Orhue, 2015, 2017; Iseghohi, Orhue, & Omage, 2017). Other effects of *D. tripetala* that have been observed by scientists have been extensively reviewed by Iseghohi (2015).

Very few toxicity studies have been carried out on this plant. Ofem, Ikpi, and Antai (2014) found that administration of the ethanolic extract of *D. tripetala* fruits at 85 mg/kg bw for 14 days can reduce bile production in normal healthy rats, even though a previous study by López‐Martín et al. (2002) showed that *D. tripetala* contains uvariopsine, an alkaloid that improves bile secretion and attenuates hepatic disorders in rats. Another attempt at evaluating the toxicity of *D. tripetala* showed that the ethanolic extract of this plant is hematotoxic to rats when administered orally at certain doses (Ikpi & Nku, 2008). Another study by Anaga, Shoyinka, and Asuzu (2006) showed that the ethyl acetate extract of *D. tripetala* roots exhibits mild toxicity on the liver, kidney, spleen, and blood cells, but is seemingly beneficial to the hearts of mice following prolonged exposure.

To the best of our knowledge, there has been no study evaluating the acute and subchronic effects of administering the aqueous and ethanolic extracts of *D. tripetala* fruits orally to rats; hence, we set out to fill this gap in knowledge. A few researchers have carried out experiments on the leaves, roots, and seeds of *D. tripetala* to determine its phytochemical, proximate, and elemental composition as well as its in vitro antioxidant properties, but to the best of our knowledge, there is no publication where the phytochemical content and in vitro antioxidant properties of the fruit of this plant were compared to its extracts. In the present paper, we therefore set out to compare the phytochemical constituents and in vitro antioxidant properties of *D. tripetala* fruit powder with its aqueous and ethanolic extracts.

## 2 METHODS

### 2.1 Plant material

Ripe fruits of *D. tripetala* were harvested in Benin city, Nigeria. The fruits were sliced and sun‐dried, then ground to fine powder. Aqueous and ethanolic extracts of the fruits were prepared following the method of Iseghohi and Orhue (2017), with the ethanolic extract made by soaking the plant powder in ethanol rather than water. For administration to animals, each of the resulting extracts (concentrated with a freeze dryer) was suspended in distilled water. For phytochemical analyses and in vitro antioxidant assays, stock solutions of the plant powder, aqueous extract, and ethanolic extract were prepared by soaking the respective plant samples in a solvent (depending on the assay) at a concentration of 100 mg/ml for 2 hr with regular shaking. The supernatants obtained were subjected to qualitative and quantitative phytochemical analysis as well as in vitro antioxidant activities.

### 2.2 Qualitative phytochemical analysis

The following phytochemicals were screened for phenols, flavonoids, saponins, tannins, and alkaloids. Standard methods of Trease and Evans (1989), Harborne (1998), and Sofowora (1993) were used.

### 2.3 Quantitative phytochemical analysis

The methods used for quantitative determination of the phytochemicals we screened for are as follows: phenols (Senguttuvan, Paulsamy, & Karthika, 2014), flavonoids (Senguttuvan et al., 2014), saponins (Senguttuvan et al., 2014), tannins (Osuagwu & Eme, 2013), and alkaloids (Senguttuvan et al., 2014). In all cases, the plant powder was soaked in water prior to the experiment, as described in the “Plant material” section above.

### 2.4 In vitro antioxidant assays

#### 2.4.1 DPPH scavenging activity

The method of Shen et al. (2010) with slight modifications was used. Rutin was used as standard; 1 mg/ml of plant extracts and standard were diluted to a range of concentrations (10–700 μg/ml in methanol). 0.2 mM solution of DPPH in methanol was prepared. 500 μl of this DPPH was collected and added to the different concentrations of 3 ml of plant extracts and standard. This was followed by vigorous shaking. The mixture was allowed to stand for 30 min at room temperature. A control was prepared using methanol for baseline correction. The absorbance was measured at 517 nm. Lower absorbance indicated higher radical scavenging activity**.** The formula for calculating DPPH radical scavenging activity (% inhibition) is: % inhibition = (A_0_ − A_1_)/A_0_) × 100, where A_0_= absorbance of blank and A_1_= absorbance of sample or standard.

#### 2.4.2 Hydrogen peroxide scavenging activity

The method described by Okolie et al. (2014) with slight modifications was used. Rutin was used as standard. The procedure is as follows: PBS (pH 7.4) was prepared; 1 mg/ml plant extract and standard were diluted to different concentrations (10–700 μg/ml) in methanol. Peroxide (20 mM) was diluted in PBS. To 2 ml of plant extract or standard, 4 ml of the solution of H_2_O_2_ and PBS was added. The mixture was allowed to stand for 10 min. Absorbance was taken at 230 nm against blank (which contains no H_2_O_2_). Lower absorbance indicated higher radical scavenging activity. The formula for calculating hydrogen peroxide scavenging activity (% inhibition) is as follows: % inhibition = (A_0_ − A_1_)/A_0_) × 100, where A_0_ = absorbance of blank and A_1_ = absorbance of sample or standard**.**

### 2.5 Ferric reducing power

The method described by Okolie et al. (2014) with slight modifications was used. Rutin was used as a standard. The procedure is described as follows: The following solutions were prepared: 1% K_3_Fe(CN)_6_, 0.1% FeCl_3_ (fresh), TCA (10%), and phosphate buffer (pH 6.6). The plant samples and standard were diluted from 1 μg/ml to (10–700 μg/ml). To 1 ml of plant extract or standard, 2.5 ml of phosphate buffer (pH 6.6) and 2.5 ml of K_3_Fe(CN)_6_ were added. The mixture was incubated at 50°C for 20 min. This was followed by the addition of 2.5 ml of TCA and centrifugation at 3,000 *g* for 10 min. 2.5 ml of liquid was drawn from upper layer. 2.5 ml of distilled water was added to the upper layer retrieved. 0.5 ml of FeCl_3_ (freshly prepared) was added, and the absorbance was measured at 700 nm. A graph of concentration versus absorbance was plotted. Higher absorbance values indicated a higher ferric reducing power.

### 2.6 Animals

Male albino rats of Wistar strain weighing an average of 160 ± 20 g were used for this study. The animals were housed in wooden cages sealed with barbed wire. They were allowed to acclimatize for 2 weeks and were fed with pellets and given tap water to drink. The comfort of the animals was taken into special consideration throughout the duration of the experiment. During the experiments, the route of administration of the plant extract was oral (using a gavage). All experiments on the animals were carried out in accordance with the NIH guide for the care and use of laboratory animals (NIH publication No. 8023, revised 1978) and were approved by the committee for the ethical use of laboratory animals, Faculty of Life sciences, University of Benin, Nigeria.

### 2.7 Experimental design

Two kinds of toxicity studies were carried out: acute toxicity and subchronic toxicity study. To determine the acute toxicity of *D. tripetala* extracts, we used the method of Lorke (1983). To determine the subchronic toxicity of these extracts, we randomized 18 rats into three groups of six rats each (groups A–C). Group A served as control and the rats in this group were only given feed and water. Groups B and C were given 1,000 mg/kg bw of aqueous and ethanolic extract of *D. tripetala,* respectively, for 28 days. This dose was chosen since the animals survived the acute administration of a dose that is five times higher than this. After the 28‐day period, the animals were sacrificed and blood was collected for biochemical assays. The liver, kidney, and heart were also collected and used for histopathological and biochemical analysis.

### 2.8 Biochemical assays

The serum obtained was subjected to the following assays: AST, ALT, ALP, GGT, total cholesterol, triglyceride, total protein, albumin, globulin, urea, creatinine, SOD, catalase, and MDA. All but four assays were carried out using kits from Randox Laboratories (the United Kingdom), and the manufacturer's protocol was followed in all cases. SOD, catalase, and MDA assays were carried out using reagents purchased from Pyrex Laboratories, Benin city, Nigeria. The methods of Misra and Fridovich (1972), Goth (1991), and Buege and Aust (1978) were used for SOD, catalase, and MDA assays, respectively. ALP was carried out using Teco Diagnostic Kit (the manufacturer's protocol was also followed). The chemicals used for phytochemical analysis were purchased from Pyrex Chemicals Limited, Benin city, Nigeria. All reagents used were of analytical grade.

### 2.9 Histopathology

Portions of the liver, kidney, and heart were fixed in 10% neutral buffered formalin for histopathological analysis. A Leica TP2010 automatic tissue processor was used to process the tissues, which were stained with hematoxylin and eosin and viewed under a microscope using 10× and 40× magnification.

### 2.10 Statistical analysis

Statistical analysis (one‐way ANOVA followed by Tukey's post hoc test) was carried out using GraphPad Prism version 7.0. *p* values less than 0.05 were taken as significant. Experiments were performed in triplicate, and data are presented as mean ± SEM.

## 3 RESULTS

### 3.1 Qualitative phytochemical analysis

The results of the phytochemical screening of the powder and extracts of ripe *D. tripetala* fruits are shown in Table 1. Phenols, flavonoids, saponins, tannins, and alkaloids were detected in the plant powder and the extracts.

**Table 1:** Phytochemical content of ripe *Dennettia tripetala* fruits

	Plant powder	Aqueous extract	Ethanolic extract
Phenols	**++**	**+**	**+**
Flavonoids	**++**	**+**	**+**
Saponins	**++**	**+**	**++**
Tannins	**++**	**+**	**+**
Alkaloids	**+**	**+**	**+**

*Note*

+ means the phytochemical is present to a moderate extent. ++ means the phytochemical is present to a high extent.

### 3.2 Quantitative phytochemical analysis

The results of the quantitative phytochemical analysis of the powder and extracts of ripe *D. tripetala* fruits are shown in Table 2. The results show that the plant powder had significantly higher content of all the phytochemicals tested when compared to the aqueous and ethanolic extracts. The aqueous extract had a slightly higher content of phenols, flavonoids, and alkaloids than the ethanolic extract, while the ethanolic extract had a slightly higher content of saponins and tannins than the aqueous extract.

**Table 2:** Quantitative phytochemical analysis of ripe *Dennettia tripetala* fruits

Phytochemical	Plant powder	Aqueous extract	Ethanolic extract
Phenols (mg RE/g)	3.276 ± 0.011^a^	3.092 ± 0.068^b^	2.976 ± 0.072^b^
Flavonoids (mg RE/g)	1.176 ± 0.109^a^	0.943 ± 0.003^b^	0.811 ± 0.023^b^
Saponins (mg DE/g)	0.561 ± 0.021^a^	0.423 ± 0.012^b^	0.557 ± 0.024^a^
Tannins (mg TE/g)	2.214 ± 0.028^a^	2.086 ± 0.014^b^	2.091 ± 0.017^b^
Alkaloids (%)	0.475 ± 0.031^a^	0.350 ± 0.027^b^	0.340 ± 0.013^b^

*Note*

Results are in mean ± SEM in triplicate. Values on the same row with different superscripts differ from one another significantly (*p* < 0.05). Values on the same row with same superscript do not differ significantly. RE means rutin equivalent. DE means diosgenin equivalent. TE means tannic acid equivalent. The units are in mg of standard phytochemical per gram of starting plant sample.

### 3.3 In vitro antioxidant activity

**Figure 1:**
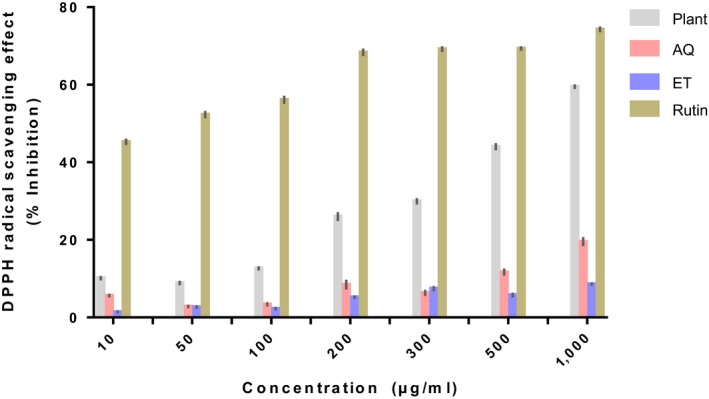
DPPH radical scavenging capacity of extracts of ripe Dennettia tripetala fruits. Plant refers to the plant powder. AQ is the aqueous extract of *Dennettia tripetala*. ET is the ethanolic extract of *D. tripetala*. Rutin was used as standard

**Figure 2:**
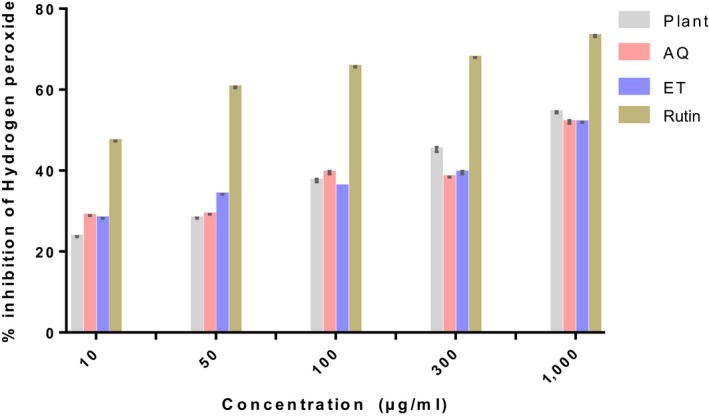
Hydrogen peroxide scavenging capacity of ripe Dennettia tripetala fruits. Plant refers to the plant powder. AQ is the aqueous extract of *Dennettia tripetala*. ET is the ethanolic extract of *D. tripetala*. Rutin was used as standard

**Figure 3:**
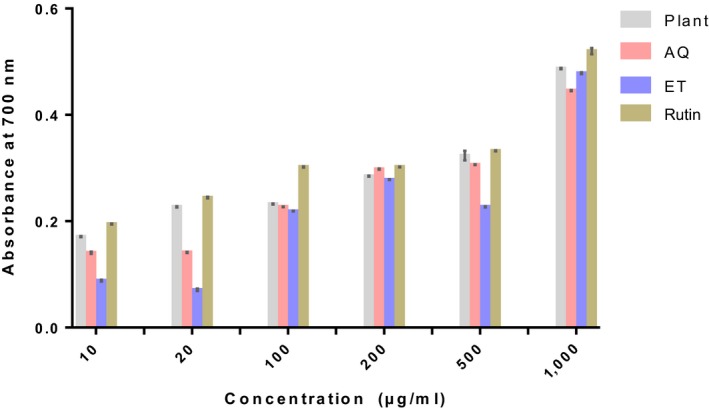
Ferric reducing capacity of ripe Dennettia tripetala fruits. Plant refers to the plant powder. AQ is the aqueous extract of *Dennettia tripetala*. ET is the ethanolic extract of *D. tripetala*. Rutin was used as standard

The results of the in vitro antioxidant capacity of the powder and extracts of ripe *D. tripetala* fruits are depicted in Figures 1, 2, 3.

#### 3.3.1 DPPH radical scavenging activity

The plant powder had the highest ability to scavenge DPPH radicals at all the concentrations tested even though it was not as effective as the standard compound, rutin (Figure 1). The aqueous extract was significantly (*p* < 0.001) more effective than the ethanolic extract at the two highest doses as well as at the lowest dose tested.

#### 3.3.2 Hydrogen peroxide scavenging activity

The plant powder exhibited the lowest hydrogen peroxide scavenging capacity at the two lowest doses, but at the two highest doses, the plant powder exhibited the highest hydrogen peroxide scavenging capacity although it was not as impressive as the standard, rutin (Figure 2). The ethanolic extract exhibited a significantly (*p* < 0.001) higher scavenging activity than the aqueous extract at a dose of 50 μg/ml, but when the dose was increased to 100 μg/ml, the aqueous extract exhibited a significantly (*p* < 0.001) higher scavenging potential. At the other doses, there was no difference between the scavenging potentials of both extracts.

#### 3.3.3 Ferric reducing power

The plant powder had the highest ability to reduce ferric ions although it was not as impressive as the standard, rutin (Figure 3). The aqueous extract had a significantly (*p* < 0.01) higher potential to reduce ferric ions than ethanolic extract at all doses except the highest dose where the ethanolic extract showed a significantly (*p* < 0.001) higher reducing power than the aqueous extract.

### 3.4 Acute toxicity of *Dennettia tripetala*

During the two phases of this study, there was no mortality recorded in any of the rats. Also, there was weight gain over the 2 week observation period. We also did not observe any strange behavior in the animals that could have indicated toxicity. There was also no damage observed upon macroscopic examination of the organs of the animals. In summary, the highest dose of 5,000 mg/kg bw of the aqueous and ethanolic extracts of *D. tripetala* fruits was well tolerated by the rats; the LD_50_ is therefore greater than 5,000 mg/kg bw.

### 3.5 Subchronic toxicity of *Dennettia tripetala*

The aqueous and ethanolic extracts of *D. tripetala* fruits did not significantly alter the serum activities of AST, ALT, ALP, and GGT in the rats tested (Table 3). The aqueous and ethanolic extracts of *D. tripetala* fruits also did not significantly alter the serum cholesterol level even though the triglyceride level reduced by about 50% in the rats administered with *D. tripetala* extracts (Table 4). The extracts did not significantly alter the liver triglyceride level even though the cholesterol level increased by an average of nearly 50% in the rats administered with *D. tripetala* extracts (Table 4). The aqueous and ethanolic extracts of *D. tripetala* fruits did not significantly alter the serum protein profile (Table 5) nor did they significantly alter the antioxidant enzyme activity and lipid peroxidation status in the liver of the rats tested (Table 6). The extracts also did not significantly alter the concentration of urea and creatinine in the serum (Table 7) nor did they significantly alter the activity of superoxide dismutase or the lipid peroxidation status in the kidney of the rats tested, but the aqueous and ethanolic extracts were found to cause significant increases (*p* < 0.01 and *p* < 0.05, respectively) in the activity of catalase in the kidney of the rats tested (Table 7).

**Table 3:** Effect of *Dennettia tripetala* on liver marker enzymes in the serum of rats

Groups	AST (U/L)	ALT (U/L)	ALP (U/L)	GGT (U/L)
Control	106.1 ± 7.78^a^	29.33 ± 8.99^a^	53.92 ± 4.27^a^	476.8 ± 16.79^a^
AQDT 1000	111.5 ± 5.29^a^	35.33 ± 4.06^a^	49.61 ± 4.64^a^	453.8 ± 9.98^a^
ETDT 1000	101.7 ± 7.62^a^	35.00 ± 5.80^a^	49.10 ± 4.11^a^	432.4 ± 50.04^a^

*Note*

AQDT refers to the aqueous extract of *D. tripetala*. ETDT refers to the ethanolic extract of *D. tripetala*. 1,000 refers to the dose of the extract (mg/kg b.w). The values presented are the mean ± SEM. *n* = 6. Values with the same superscript are not significantly different from one another.

**Table 4:** Effect of *Dennettia tripetala* on serum and liver lipid profile of rats

Groups	Serum total CHOL (mg/dl)	Serum TAG (mg/dl)	Liver total CHOL (mg/dl)	Liver TAG (mg/dl)
Control	60.34 ± 4.91^a^	227.5 ± 18.67^a^	124.6 ± 33.98^a^	506.1 ± 29.91^a^
AQDT 1000	71.30 ± 5.21^a^	108.8 ± 37.32^a^	252.0 ± 109.8^a^	499.5 ± 48.42^a^
ETDT 1000	67.88 ± 9.99^a^	107.9 ± 21.39^a^	221.7 ± 59.40^a^	444.8 ± 29.44^a^

*Note*

AQDT refers to the aqueous extract of *D. tripetala*. ETDT refers to the ethanolic extract of *D. tripetala*. 1,000 refers to the dose of the extract (mg/kg b.w). The values presented are the mean ± SEM. *n* = 6. Values with the same superscript are not significantly different from one another.

**Table 5:** Effect of *Dennettia tripetala* on serum protein profile of rats

Groups	Total protein (g/dl)	Albumin (g/dl)	Globulin (g/dl)	Albumin: Globulin ratio
Control	6.86 ± 0.23^a^	3.92 ± 0.24^a^	3.34 ± 0.46^a^	1.36 ± 0.21^a^
AQDT 1000	7.11 ± 0.14^a^	3.28 ± 0.02^a^	3.73 ± 0.07^a^	0.91 ± 0.03^a^
ETDT 1000	7.10 ± 0.16^a^	3.72 ± 0.08^a^	3.50 ± 0.18^a^	0.92 ± 0.10^a^

*Note*

AQDT refers to the aqueous extract of *D. tripetala*. ETDT refers to the ethanolic extract of *D. tripetala*. 1,000 refers to the dose of the extract (mg/kg b.w). The values presented are the mean ± SEM. *n* = 6. Values with the same superscript are not significantly different from one another.

**Table 6:** Effect of *Dennettia tripetala* on antioxidant enzyme activity and lipid peroxidation status in liver of rats

Groups	SOD (units/g wet tissue)	Catalase (units/g wet tissue)	MDA (units/g wet tissue)
Control	183.3 ± 10.54^a^	4,974 ± 8.31^a^	0.14 ± 0.02^a^
AQDT 1000	206.7 ± 16.67^a^	5,035 ± 20.98^a^	0.13 ± 0.01^a^
ETDT 1000	211.3 ± 30.09^a^	5,062 ± 49.29^a^	0.11 ± 0.01^a^

*Note*

AQDT refers to the aqueous extract of *D. tripetala*. ETDT refers to the ethanolic extract of *D. tripetala*. 1,000 refers to the dose of the extract (mg/kg b.w). The values presented are the mean ± SEM. *n* = 6. Values with the same superscript are not significantly different from one another.

**Table 7:** Effect of *Dennettia tripetala* on serum urea and creatinine concentration and the antioxidant enzyme activity and lipid peroxidation status in the kidney of rats

Groups	Urea (mg/dl)	Creatinine (mg/dl)	SOD (units/g wet tissue)	Catalase (units/g wet tissue)	MDA (units/g wet tissue)
Control	22.07 ± 0.72^a^	8.13 ± 0.64^a^	333.3 ± 88.19^a^	9,540 ± 9.00^a^	0.22 ± 0.03^a^
AQDT 1000	29.00 ± 4.86^a^	7.11 ± 1.44^a^	400.0 ± 81.65^a^	9,639 ± 17.23^b^**	0.21 ± 0.01^a^
ETDT 1000	29.97 ± 5.43^a^	8.04 ± 0.45^a^	466.7 ± 133.3^a^	9,627 ± 13.08^b^*	0.20 ± 0.01^a^

*Note*

AQDT refers to the aqueous extract of *D. tripetala*. ETDT refers to the ethanolic extract of *D. tripetala*. 1,000 refer to the dose of the extract (mg/kg b.w). The values presented are the mean ± SEM. *n* = 6. Values with the same superscript are not significantly different from one another. Values with different superscripts are significantly different from one another (*p < 0.05, *^*^ *p* < 0.01).

### 3.6 Histology

**Figure 4:**
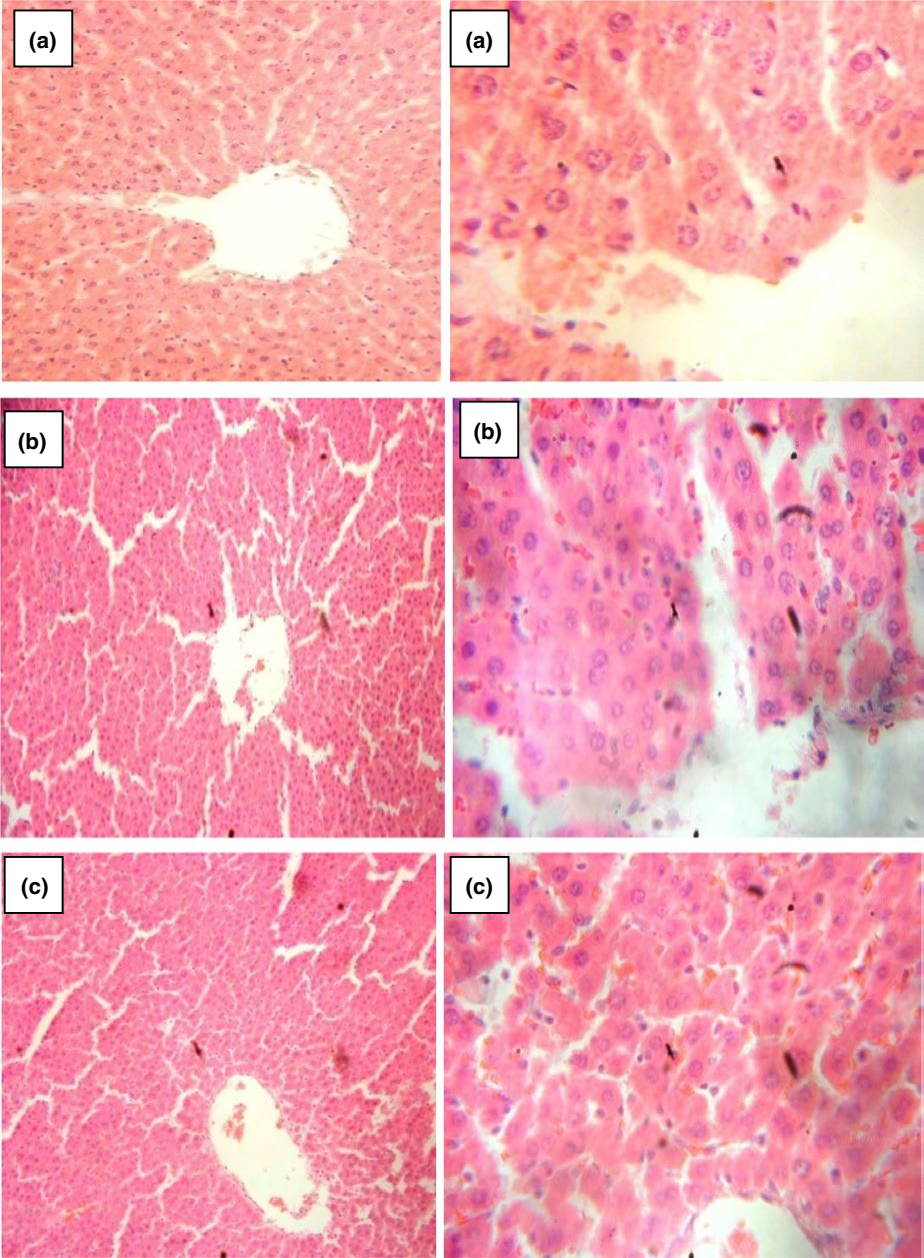
Sections from the liver of experimental rats. Sections were stained with H/E and viewed using a 10× objective (sections on the left) and 40× objective (sections on the right). The centriole of the liver in Fig B and C was found to have a few congested areas, but otherwise, the liver histology looks normal overall

**Figure 5:**
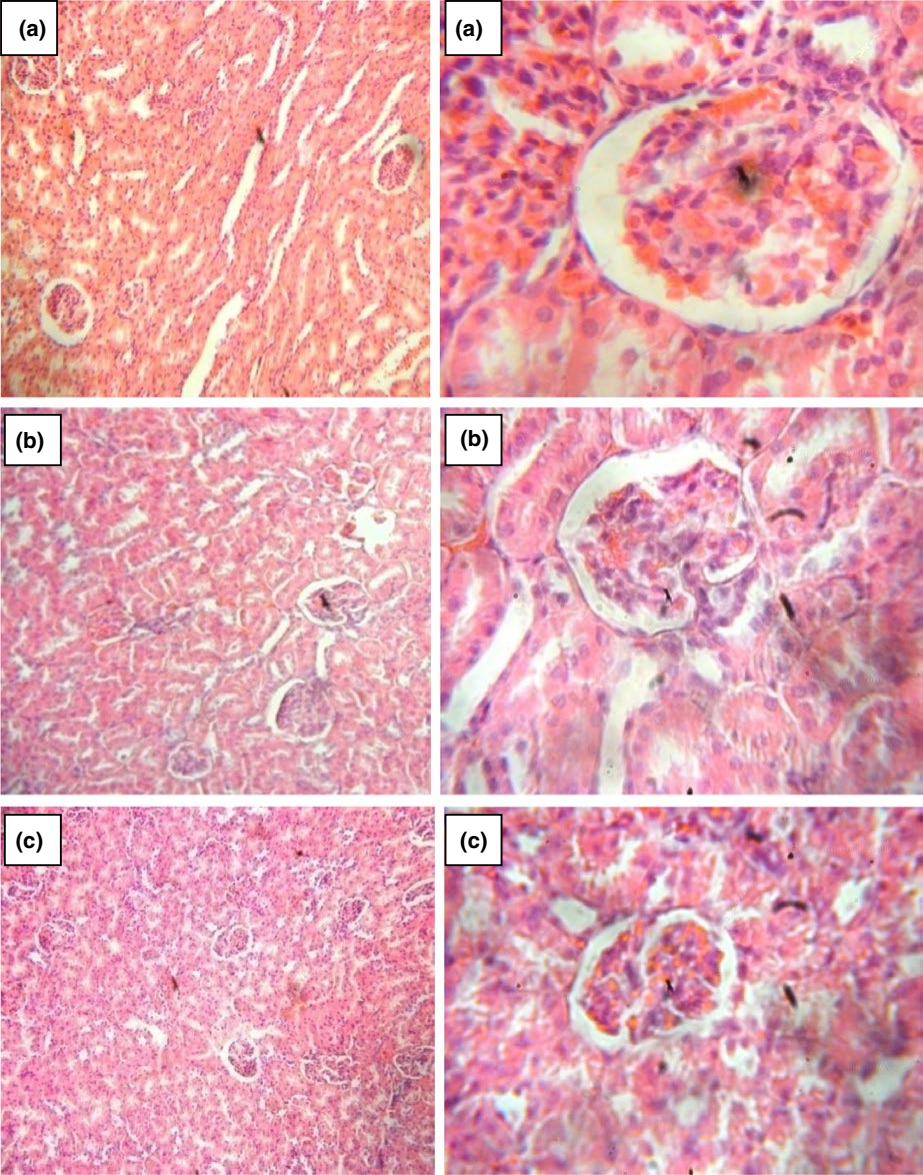
Sections from the kidneys of experimental rats. Sections were stained with H/E and viewed using a 10× objective (sections on the left) and 40× objective (sections on the right). All sections show normal kidney histology

**Figure 6:**
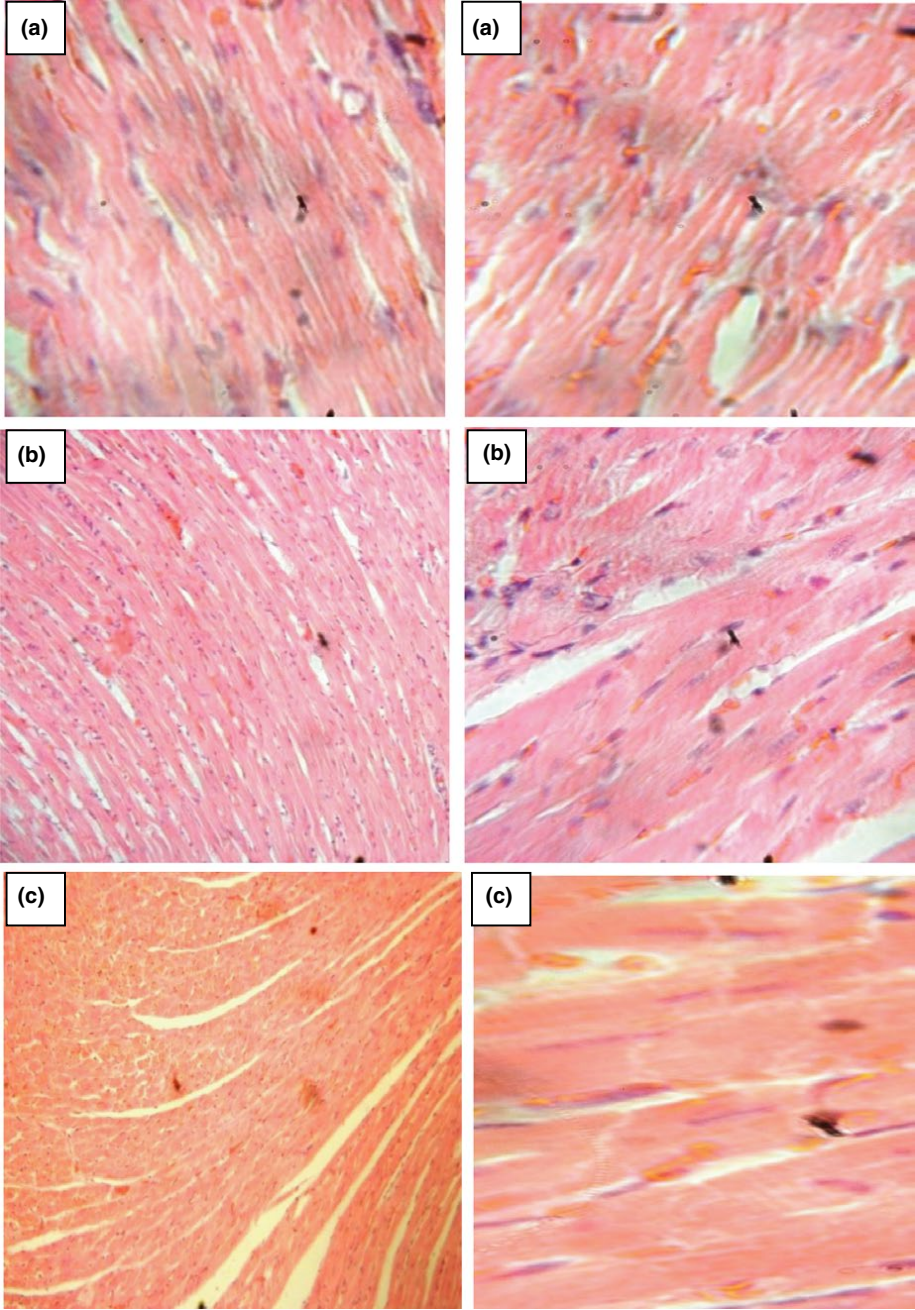
Sections from the heart of experimental rats. Sections were stained with H/E and viewed using a 10× objective (sections on the left) and 40× objective (sections on the right). All sections show normal heart histology

Images showing the effect of *D. tripetala* aqueous and ethanolic extracts on the liver, kidney, and heart are shown in Figures 4, 5, 6. The results show that the aqueous and ethanolic extracts of *D. tripetala* caused a negligible amount of congestion in the centrioles of the liver, but the extracts were not in any other way toxic to the liver, kidney, or heart of the rats.

## 4 DISCUSSION

### 4.1 Plants studies

*Dennettia tripetala* is not only eaten for its spicy nature but also for its medicinal value. Our results show the presence of phenols, flavonoids, saponins, tannins, and alkaloids in ripe *D. tripetala* fruit powder and in its aqueous and ethanolic extracts. Alkaloids have been used in the manufacture of steroidal drugs (Maxwell, Seepersaud, Pingal, Mootoo, & Reynolds, 1995). Alkaloids are also used as analgesics, antibiotics, and antispasmodics (Osuagwu & Eme, 2013). Phenolic compounds tend to inhibit the growth of pathogens (Osuagwu & Eme, 2013). They therefore have antiseptic applications. They are also antioxidants and have been shown to have anticancer activity (Osuagwu & Eme, 2013). Phenolic compounds are a large group of phytochemicals that include flavonoids, phenolic acids, tannins, coumarins amongst others (Omage & Azeke, 2014). Saponins are known to have hypotensive and cardiac depressant properties (Omage & Azeke, 2014). Saponins help to reduce hypercholesterolemia (Soetan & Aiyelaagbe, 2009). Apart from serving as emulsifiers, saponins also act as expectorants (Edeoga, Okwu, & Mbaebie, 2005). Saponins can also induce hemolysis and act as antifungal agents (Osuagwu & Eme, 2013).

Flavonoids are potent antioxidants, with the ability to inhibit tumor progression and manage free radical damage induced by toxins, viruses, and microbes amongst others (Osuagwu & Eme, 2013). Tannins have an astringent property; they can hasten wound healing as well as repair inflamed mucous membranes (Osuagwu & Eme, 2013). They can therefore heal burns and ulcers. They also induce tumor regression (Soetan & Aiyelaagbe, 2009) and act as antibiotics (Nwogu, Igwe, & Emejulu, 2008). The presence of these phytochemicals in *D. tripetala* lends credence to the use of this plant for the management of diseases.

The phytochemicals present in the *D. tripetala* fruit powder and extracts used in this study are likely to be responsible for the observed in vitro antioxidant properties in this study. In this study, *D. tripetala* fruit powder and extracts displayed the ability to bleach DPPH, scavenge hydrogen peroxide, and reduce ferric ions. The DPPH results showed that the aqueous extract had a higher antioxidant potential than the ethanolic extract especially at higher doses, and the plant powder itself had the highest antioxidant potential, although it was quite lower than the standard, rutin.

Results from the present study also showed that aqueous and ethanolic extracts of *D. tripetala* fruits have a similar ability to scavenge hydrogen peroxide while the plant powder has a higher ability, albeit still much lower than rutin. The aqueous and ethanolic extracts of *D. tripetala* fruits also have a similar ability to reduce ferric ions to ferrous ions even though the ethanolic extract is more effective at higher doses and the aqueous extract, more effective at lower doses. Still, the plant powder is better at all doses and almost as good as the standard, rutin.

In summary, our result shows that the aqueous extract has a higher DPPH scavenging activity than the ethanolic extract, and both extracts have similar abilities to scavenge hydrogen peroxide and ferric ions. Our results can be explained by the fact that the aqueous extract of *D. tripetala* fruits used in this study contains a higher amount of flavonoids, phenols, and alkaloids than the ethanolic extract, and these phytochemicals may be partly responsible for the higher antioxidant activity elicited by the aqueous extract, at least in the DPPH assay.

### 4.2 Animal studies

The fruits of *D. tripetala* are consumed by humans; therefore, it was not surprising that the aqueous and ethanolic extracts of *D. tripetala* used in this study were not acutely toxic to rats even when consumed at a high dose of 5,000 mg/kg bw. A group of investigators have previously tried to ascertain the acute toxicity of the ethanolic extract of *D. tripetala* fruits on mice. They found that the LD_50_ of *D. tripetala* was 251.19 mg/kg bw when administered intraperitoneally to normal healthy mice (Ikpi & Nku, 2008). Although this value is far different from ours (which is greater than 5,000 mg/kg bw), it should be noted that these investigators used mice, which are far smaller than rats; this may be a reason for the huge difference in the results obtained. The route of administration of the extract was also different from ours, and this may possibly be a reason for the variation in the results obtained.

Anaga et al. (2006) also investigated the acute toxicity of the ethyl acetate extract of *D. tripetala* roots on mice. They got an LD_50_ value of 1,120 mg/kg bw. Although this value is far different from ours and that of Ikpi and Nku (2008), it should be noted that the plant part used was different, as well as the solvent for extraction. This may have greatly contributed to the differences in the values obtained by different groups of researchers, our group included.

From the results of our subchronic toxicity study, it can be concluded that the aqueous and ethanolic extracts of *D. tripetala* fruits were not harmful to the rats at the biochemical level, after 28 days of daily administration. Nonetheless, it is important to note that a 28‐day administration of *D. tripetala* extracts in rats reduced the concentration of triglyceride in the serum by approximately 50% although this was not found to be statistically significant. Another outcome of our results is that the level of cholesterol in the liver of the rats that received the extracts increased by up to 50% although it was also not also statistically significant. This observation may be explained by the hypothesis that the plant extracts may have caused slight alterations in the ability of the liver to metabolize triglyceride and cholesterol, as there are insignificant changes in the triglyceride and cholesterol concentrations of the liver as well. These results therefore point to the fact that administration of this plant extract for a prolonged period has some effects on the lipid profile of the blood. Rather than neglecting this outcome, it will be interesting to study this phenomenon further in the future.

Results from our experiment also showed that the extracts of *D. tripetala* fruits caused an increase (*p* < 0.05) in the activity of catalase in the kidneys of the rats. Interestingly, the activity of SOD (another pertinent antioxidant enzyme) also increased (*p* > 0.05) in the kidneys of the rats given the plant extracts. This could mean either of two things. The extracts may have simply induced a boost in the kidneys’ ability to deal with oxidative stress by increasing the activity of catalase and SOD, which are potent antioxidant enzymes, or the extracts themselves may have induced some form of stress that triggered an increase in the kidneys’ antioxidant defense. Whichever hypothesis holds true, the conclusion from the results of this study is that *D. tripetala* extracts increased the ability of certain cells of the body to combat oxidative stress by increasing the activities of catalase and SOD.

Histopathological images from the present study showed that the aqueous and ethanolic extracts of *D. tripetala* caused a negligible amount of congestion in the centrioles of the liver but were not in any other way toxic to the liver, kidney, or heart of the rats. The slight congestion caused by the extracts to the centriole of the liver has been previously observed by our research group where the extract in an attempt to clear steatosis caused by the hepatorenal toxicant carbon tetrachloride induced a sort of “side effect” which featured patchy congestions of the centriole of the liver (Iseghohi & Orhue, 2017). In conclusion, a 28‐day administration of *D. tripetala* extracts does not cause considerable alterations to the organs and cells of normal healthy rats. As for the slight alterations in the liver's ability to metabolize lipids, this was not severe enough to warrant caution, although it will be interesting to look into this more closely in future studies.

## 5 CONCLUSION

The powder, aqueous, and ethanolic extracts of *D. tripetala* fruits possess phytochemicals with proven medicinal properties. The powder from the fruit has the highest content of these phytochemicals and the best antioxidant properties in vitro while both extracts have very similar content of the phytochemicals as well as similar antioxidant properties in vitro. These findings therefore validate some of the ethnomedicinal uses of the fruits of *D. tripetala*. Our findings also show that it is safe to consume *D. tripetala* fruits as a source of nutrients or for medicinal purposes, for a prolonged period of time as the side effects we observed in rats were not serious enough to warrant caution.

## ETHICAL REVIEW

The protocols used in this study were reviewed and approved by the Animal Ethics Committee of the Faculty of Life sciences, University of Benin, Nigeria.

## CONFLICT OF INTEREST

The authors declare that they do not have any conflict of interest.
